# The importance of sediments in ecological quality assessment of stream headwaters: embryotoxicity along the Nidda River and its tributaries in Central Hesse, Germany

**DOI:** 10.1186/s12302-018-0150-4

**Published:** 2018-06-20

**Authors:** Mona Schweizer, Andreas Dieterich, Núria Corral Morillas, Carla Dewald, Lukas Miksch, Sara Nelson, Arne Wick, Rita Triebskorn, Heinz-R. Köhler

**Affiliations:** 1 Animal Physiological Ecology, Eberhard Karls University of Tübingen, Auf Der Morgenstelle 5, 72076 Tübingen, Germany; 2 Faculty of Biology, University of Barcelona, Diagonal, 643, 08028 Barcelona, Spain; 3 The German Federal Institute of Hydrology (BfG), Am Mainzer Tor 1, 56068 Koblenz, Germany; 4 Steinbeis Transfer-Center for Ecotoxicology and Ecophysiology, Blumenstr. 13, 72108 Rottenburg am Neckar, Germany

**Keywords:** FET, Sediment toxicity, Ecosystem health, Anthropogenic impacts

## Abstract

**Background:**

Although the crucial importance of sediments in aquatic systems is well-known, sediments are often neglected as a factor in the evaluation of water quality assessment. To support and extend previous work in that field, this study was conducted to assess the impact of surface water and sediment on fish embryos in the case of a highly anthropogenically influenced river catchment in Central Hesse, Germany.

**Results:**

The results of 96 h post fertilisation fish embryo toxicity test with *Danio rerio* (according to OECD Guideline 236) revealed that river samples comprising both water and sediment exert pivotal effects in embryos, whereas surface water alone did not. The most prominent reactions were developmental delays and, to some extent, malformations of embryos. Developmental delays occurred at rates up to 100% in single runs. Malformation rates ranged mainly below 10% and never exceeded 25%.

**Conclusion:**

A clear relationship between anthropogenic point sources and detected effects could not be established. However, the study illustrates the critical condition of the entire river system with respect to embryotoxic potentials present even at the most upstream test sites. In addition, the study stresses the necessity to take into account sediments for the evaluation of ecosystem health in industrialised areas.

## Background

This study was conducted within the joint project NiddaMan, which focused on diagnosis of ecosystem health in the catchment of the Nidda River in Hesse, Germany, as a scientific basis for river management. The Nidda catchment, including the Nidda River and its tributaries Horloff and Usa, can be regarded as a model for medium-sized stream systems influenced by intense industrial and agricultural activity in modern industrialised countries. With regard to the European Water Framework Directive (2000/60/EC) surface waters were supposed to be in a good ecological state until 2015. As it is well-known, however, that many of those German water bodies had fallen far short of this goal, therefore, the stated period was prolonged until 2027. In Germany, only 7.9% of surface waters achieved a ‘good’ and just 0.3% a ‘very good’ ecological state at the first deadline in 2015 [1]. The ecological status assessment is based on two main pillars: physicochemical parameters and biological monitoring. Physicochemical parameters can be measured continuously with relatively little effort. Data obtained are collated with existing environmental quality standards (EQS) but also may contribute to deriving new EQS for substances uncovered, yet. The clearest shortcoming of chemical analyses is that only substances tested for can be detected and quantified. But thousands of substances enter our waterbodies on a daily basis, and in the European Union alone, thousands of new ones are registered in REACH [Regulation (EC) No. 1907/2006 concerning the Registration, Evaluation, Authorisation and Restriction of Chemicals] every year. Thus, analyses of chemical compounds in surface waters are restricted to lead substances that can only give a scattered picture of the actual situation. Biological monitoring, in contrast, offers a clear snapshot of the situation in reality, but is time consuming and cost-intensive. Therefore, biomonitoring is conducted less frequently including a lower number of investigated sites. But even if biomonitoring is repeated in shorter intervals at more sites, the context between chemical measurements and status quo of biota in the field will still be lacking. Effect-based bioassays and biomarker are able to bridge that gap because they reflect the ecotoxicological potential of the studied system, may be conducted on different levels (cells, organs, organisms) and are financially feasible. As a consequence, the idea of complementing the European Water Framework Directive (WFD) with additional biotests gains proponents in the scientific community, in recent years (e.g. [2–5]).

The WFD mainly focusses on water quality but as a major driver in the source–sink dynamics of surface water systems, the influence of sediments has to be considered necessarily [6, 7]. Otherwise, analyses will lead to false evaluations of the ecotoxicological potential of those systems [8–12] and therefore, impair the achievement of the ecological goals set by the WFD [13]. In particular, fish are dependent on sediments during their entire embryonic and larval development. Sediments serve as spawning substrate and thus, have the potential to influence reproductive behaviour, hatching success, developmental processes and growth [14].

The objective of this study was to evaluate developmental toxicity of native river samples on fish embryos. To account for the effects that may be induced by particle bound substances accumulating in sediments, fish embryos were exposed to samples, either containing surface water only or surface water in combination with sediment from the respective field sites. The tests covered lethal as well as sublethal endpoints and were conducted with the zebrafish (*Danio rerio* [Hamilton 1822]) due to its simple handling, lack of a particular spawning season and the standardised test procedure (see e.g. [15, 16]).

## Methods

### Sampling location

The River Nidda and its tributaries Horloff and Usa are located in Central Hesse, Germany. They can be regarded as a very characteristic catchment system for Central Europe as they are highly anthropogenically influenced by waste water treatment plants (in the following abbreviated with WWTP) and industrial discharges, as well as by agriculture and renaturation measures. The Nidda has its source in the Vogelsberg, a low mountain range of volcanic origin in East Hesse, and enters the River Main near Frankfurt. With a length of 89.7 km, the Nidda counts among the most important water bodies in Hesse. Along its course, agriculture prevails but also grassland, settlements and industry occurs, whereas the fraction of uncultivated land is rather small and, therefore, the entire catchment is characterised by very intense land-use [17].

### Sampling sites

**Fig. 1:**
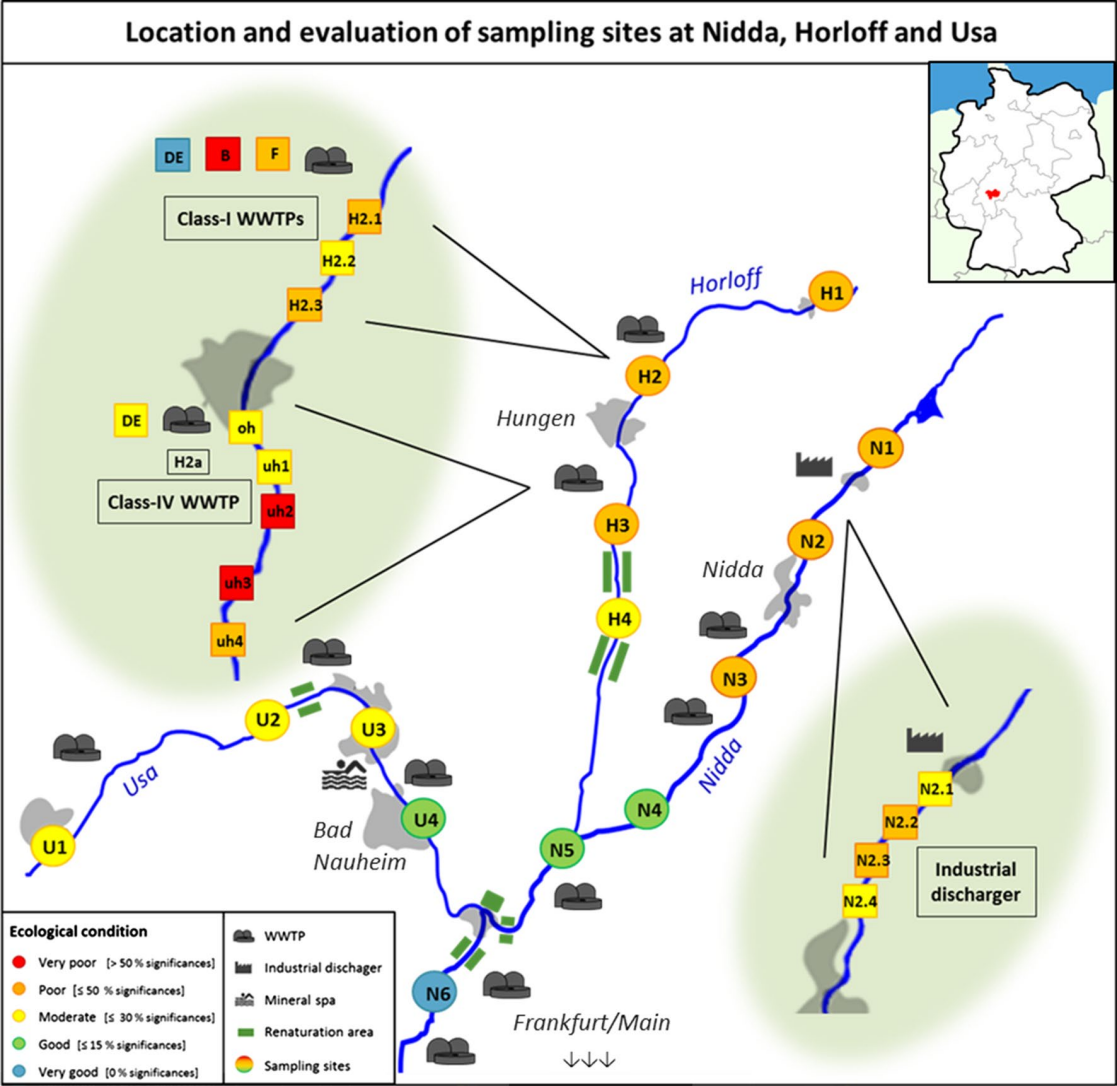
Map of the sampling area in Central Hesse, Germany including locations of sampling sites and their evaluation based on the percentage of significant endpoints compared to control treatments, as well as prominent points of discharge. Round tags mark the main sampling sites, square tags the additional sites, including the samples taken from the efflux pipes (DE), the basin (B) and the zone of effluent and river mixture (F)

The 14 main sampling sites from a first and the 16 additional sampling sites from a second sampling campaign are illustrated in Fig. 1. The sampling sites N1 and H2 were sampled in both sampling campaigns.

From the most upstream sampling site (N1) downstream the River Nidda dam as far as the most downstream sampling site (N6) northeast of Karben discharges of four communal WWTPs ranging in size from 7000 to 35,000 people equivalents (pe) and one in-house purification plant (industrial discharger) enter the river. Size classification of WWTPs in Germany is determined as followed: class-I < 1000 pe, class-II 1000–5000 pe, class-III 5001–10,000 pe, class-IV 10,001–100,000 pe and class-V > 100,000 pe. Additionally, between the fourth (N4) and fifth (N5) sampling site, the Horloff flows into the Nidda and with it discharges from several class-I WWTPs with less than 1000 pe located upstream and a class-IV WWTP covering 78,000 pe in the middle course. Besides the impact from those WWTPs, the Horloff is particularly influenced by agriculture. Downstream the class-IV WWTP, renaturation efforts have been made and are to be continued. Upstream of N6, the Usa/Wetter system enters the Nidda. Along its course, three WWTPs and several (medical) spas discharge into the Usa and, subsequently, into the Nidda. The sampling sites at the River Nidda system were set to monitor effects of different sized and equipped WWTPs (N3, N4, N5, N6, H2, H3, U2, U4), as well as the impact of special industrial dischargers [N2; U3: (medical) spas], but also the potential positive influence of renaturation efforts (N6, H4, U3) (see also Table 1). The most upstream site of each river [N1, H1, U1] was originally thought to act as reference, respectively (see Fig. 1).

**Table 1:** Overview of sampling sites in the Nidda catchment area, including the evaluation based on obtained significances

Sampling code	Coordinates	Description	Significances (%)	Category
Nidda				
*N1*	*50°27′11″N 9°02′20″E*	*Reference site; downstream the Nidda river dam*	*37.5*	*Poor*
N2.1	50°26′40″N 9°01′57″E	Directly downstream the point of efflux of the industrial discharger	26.7	Moderate
N2.2	50°26′36″N 9°01′47″E	Approximately 250 m downstream of N2.1	40.0	Poor
N2.3	50°26′29″N 9°01′43″E	Approximately 500 m downstream of N2.1	40.0	Poor
N2.4	50°26′26″N 9°01′34″E	Approximately 700 m downstream of N2.1	20.0	Moderate
*N2*	*50°25′51″N 9°01′20″E*	*Downstream an industrial discharger*	*45.0*	*Poor*
*N3*	*50°23′09″N 8°58′25″E*	*Downstream a class-IV WWTP (35,000 pe)*	*45.0*	*Poor*
*N4*	*50°19′41″N 8°52′32″E*	*Downstream a class-III WWTP (7500* *pe)*	*10.0*	*Good*
*N5*	*50°19′08″N 8°51′28″E*	*Downstream the afflux of the tributary Horloff*	*10.0*	*Good*
*N6*	*50°16′42″N 8°47′08″E*	*Downstream a class-IV WWTP (30,000* *pe), a class-III WWTP (7000 pe), four renaturalised river sections and the afflux of the Usa/Wetter tributary*	*0.0*	*Very good*
Horloff				
*H1*	*50°31′12″N 9°02′36″E*	*Reference site; no direct dischargers upstream*	*36.7*	*Poor*
*H2*	*50°30′52″N 8°57′00″E*	*Downstream several class-I WWTPs (< 1000 pe)*	*41.7*	*Poor*
H2.1	50°30′42″N 8°56′42″E	Approximately 500 m downstream of H2	46.7	Poor
H2.2	50°30′37″N 8°56′33″E	Approximately 750 m downstream of H2	26.7	Moderate
H2.3	50°30′18″N 8°56′24″E	Approximately 1400 m downstream of H2	40.0	Poor
H2a oh	50°26′22″N 8°53′58″E	Upstream a class-IV WWTP	46.7	Poor
H2a uh1	50°26′15″N 8°53′53″E	Directly downstream the point of discharge of the class-IV WWTP (78,000 pe)	26.7	Moderate
H2a uh2	50°26′05″N 8°53′48″E	Approximately 350 m downstream H2a uh1	60.0	Very poor
H2a uh3	50°24′52″N 8°54′10″E	Approximately 2650 m downstream H2a uh1, following a conservation area	53.3	Poor
H2a uh4	50°24′40″N 8°54′01″E	Approximately 3000 m downstream H2a uh1	40.0	Poor
*H3*	*50°24′49″N 8°54′09″E*	*Downstream the class-IV WWTP (78,000* *pe)*	*40.0*	*Poor*
*H4*	*50°23′57″N 8°53′56″E*	*Between two renaturalised river sections*	*25.0*	*Moderate*
Usa				
*U1*	*50°19′01″N 8°31′26″E*	*Reference site; no direct discharger upstream*	*20.0*	*Moderate*
*U2*	*50°22′48″N 8°42′45″E*	*Downstream a class-IV WWTP (50,000* *pe)*	*20.0*	*Moderate*
*U3*	*50°21′32″N 8°44′39″E*	*Within a renaturalised area in the city centre of Bad Nauheim*	*30.0*	*Moderate*
*U4*	*50°20′09″N 8°46′16″E*	*Downstream two class-IV WWTPs (43,800/47,500* *pe), and (medical) spa water discharges*	*15.0*	*Good*

With regard to the classification scheme of the Water Framework Directive (WFD) for the ecological condition of surface waters, a five class system derived. Percent limits were set by the authors. Categories apply as followed: *very good* 0% significances, *good* ≤ 15% significances, *moderate* ≤ 30% significances, *poor* ≤ 50% significances, *very poor* > 50% significances. An endpoint would be regarded as significant if at least two out of three runs showed statistically significant differences for a single endpoint. Those significances were summed up and related to the maximum number of significances possible for one sampling site. Main sampling sites are marked in italics

*WWTP* waste water treatment plant, *pe* people equivalents

In addition, in a second sampling campaign three discharge areas were examined in greater detail. For that purpose, five sampling sites were set: one upstream the discharger, one at the point of discharge and the following three in approximately similar distances downstream, whereas the length of the transects depended on the accessibility of the river banks. The decision on which sampling sites to choose for a more detailed examination was based on results from the first sampling campaign. At the River Nidda, the area downstream the industrial discharger was studied. N1 acted as upstream control. Four new sampling sites downstream the industrial discharger but upstream N2 were established (N2.1–N2.4) (see also Table 1 and Fig. 1). At the River Horloff, two areas (H1/H2 and H2a) were of particular interest. The first was located downstream the class-I WWTPs with H1 as upstream control and H2 as point of discharge, followed by three new sites downstream (H2.1–H2.3). In addition, the efflux of the last class-I WWTP at H2 was monitored in greater detail. Water was taken directly from the efflux pipe (H2-DE) and water and sediment were collected from the basin (H2-B), where a lower flow velocity prevails and the sedimentation of substances is facilitated, as well as from the area shortly behind the pipe (H2-F), where efflux and river water mix to a minor extent. Furthermore, the class-IV WWTP further downstream the Horloff was investigated more closely. Sampling site H2a oh was set as upstream control, whereas H2a uh1 marked the point of discharge and sites H2a uh2–H2a uh4 followed downstream. Additionally, water and sediment directly from the efflux pipe of the WWTP were collected (H2a uh1-DE).

### Sampling of water and sediment

Samples from each river were taken on the same day, the whole sample set including all three (first)/two (second sampling campaign) rivers on two consecutive days, except for the third sampling event of the first sampling campaign. For this first campaign, the first, second and fourth sample sets were taken in July and November 2015, as well as in July 2016. The third sampling event was spread across January (Usa), February (Nidda) and April (Horloff) 2016, due to organisational issues and water levels. The samples from the second campaign were taken in March, June and September 2017 on 1 day each. For the sampling event in March 2017, it was not possible to obtain sediment from H2a uh4 due to high water levels and strong current.

Sediment samples were collected by hand with a stainless steel shovel and bucket from a set of different spots at the sampling site and pooled before packed as separate triplicates in aluminium foil and stored on dry ice. Only the sediment’s top layer, comprised of the first 5 cm of the riverbed, was taken as it is the part of the matrix that is in direct contact with the free water phase. Water samples were filled in glass bottles and transported in cooling boxes during the term of the sampling campaign. Physicochemical parameters, including water temperature, oxygen saturation, pH, nitrate, nitrite, ammonium, phosphate, chloride and sulphate concentrations were concomitantly determined at each site. In the laboratory, water and sediment samples were stored at 20 °C until usage.

### Maintenance of zebrafish

The eggs used in this study originated from the West Aquarium strain breeding stock of zebrafish, *Danio rerio* that has been established at the Animal Physiological Ecology Group (Tuebingen University). The fish are kept in eight 90 L and one 180 L tanks filled with filtered tap water (AE-2L water filter coming with an ABL-0240-29 activated carbon filter, 0.3 μm; *Reiser*, Seligenstadt, Germany). The water temperature was held constant at 26 ± 1 °C and the physico-chemical parameters were kept in a range of pH 7.4 ± 0.2, 8–12° German hardness, 100 ± 5% oxygen saturation and 260–350 μS/cm conductivity. Neither nitrite nor nitrate concentrations exceeded critical levels of 0.025–1 mg/L, respectively 1–5 mg/L at any time point. The zebrafish were fed three times daily with dry flake food (*TetraMin*^*®*^, *Tetra GmbH*, Melle, Germany) and kept in an artificial 12:12 h light/dark cycle with the onset of light at 7 a.m. and without any influence of natural daylight. As zebrafish are modest in keeping, the aquaria lack any further equipment except for the spawning boxes inserted the evening prior to the start of the test. Those boxes of a size of 20 × 20 × 6 cm are fitted with a wire grid on top with a mesh size big enough for the eggs to fall through and are protected from being eaten by the adults, and artificial seagrass that serves as an optical breeding stimulus. Additionally, the zebrafish were fed with frozen black mosquito larvae and/or glass worms (*Poseidon Aquakultur Freeze*, Ruppichteroth, Germany) rich in proteins prior to spawning to enhance egg production.

Spawning was induced by the onset of light at 7 a.m. Fish were left undisturbed during the spawning process for another one and a half hours.

### Experimental design

To be able to draw a confident conclusion about the importance of sediments on the development of zebrafish embryos, a pretrial with exclusively water was conducted. Water samples from the first (all main sites) and second (Horloff and Usa main sites) sampling events were used. The procedure described in the following applied to water as well as to water/sediment samples in the same way.

The experiment was conducted according to the OECD Guideline 236 [18] and adjusted based on [19]. Three days before the tests started, the frozen water and sediment samples were slowly thawed in a refrigerator. The day prior to the test, spawning boxes were inserted into the fish tanks. Additionally, a set of 3 cm glass Petri dishes for the exposure (eight per treatment) and larger plates for pre-exposure (one per treatment) were filled with sediment and water and stored in an incubator at 26 ± 1 °C over night to saturate glass. Otherwise, abated effects may occur due to binding of substances to the glass and not being bioavailable, any more. The Petri dishes for the negative control filled with artificial water (0.23 g KCl, 2.59 g NaHCO_3_, 4.93 g MgS_4_O·7H_2_O and 11.76 g CaCl_2_·2H_2_O, were prepared separately in 1 L double-distilled water, respectively; then 25 mL of each solution were added to 900 mL double-distilled water) were treated in the same way. Per treatment and negative control, one pre-exposure plate and eight test replicates (Petri dishes) were used.

At the beginning of every test, the Petri dishes were emptied and refilled with 2.5 g of sediment and 3 mL surface water, whereas the pre-exposure plates only got a refill with surface water to improve the detectability of well-developed eggs that were chosen for the tests, later. Always at 8 a.m. eggs were collected from the spawning boxes with a sieve, rinsed with tap water, checked for a fertilisation rate of at least 70% as this parameter is stated as validity criterion in the OECD Guideline 236, and transferred into the pre-exposure plates. The eggs were then incubated in an incubator at 26 ± 1 °C for 2 h. Subsequently, well developed eggs of the same age, with a time point of fertilisation at 8 a.m. (≙ 0 h post fertilisation [hpf]) were randomly picked from the pre-exposure plates and transferred into the prepared small Petri dishes. For the second sampling campaign we used three eggs per Petri dish, as samples were tested as a set per river, thus to avoid artefacts that would have resulted from temporal delays within the process of embryo observation, we decided to reduce the number of eggs to be able to keep the number of eight replicates consistent. The total number of individuals per treatment was 32 for the pre- and main trial and 24 for the second sampling campaign. The dishes were stored in an incubator at 26 ± 1 °C and with an artificial 12:12 light/dark cycle. The embryonic development was observed under a stereo microscope (*Stemi 2000*-*C*, *Zeiss*) at defined time points (12, 24, 48, 60, 72 and 96 hpf) and checked for lethal and sublethal endpoints including mortality, hatching success, heart rate, developmental delays and malformations (see Table 2). Heart beat was counted for 20 s in two randomly chosen embryos per Petri dish and extrapolated to beats per minute. Coagulated eggs and egg shells from hatched larvae were removed from the dishes to avoid a decrease in oxygen concentration due to the degradation of biological material. Unlike the way of proceeding in OECD Guideline 236, embryos lacking somite formation were regarded as developmentally retarded and kept in the experiment even after 48 hpf, as we have observed such embryos to proceed with their development. The test was performed in consecutive triplicates (three runs per sampling event and site).

**Table 2:** Overview of observed lethal and sublethal endpoints at the respective time points

Endpoint	12 hpf	24 hpf	48 hpf	60 hpf	72 hpf	96 hpf
Mortality	X	X	X	X	X	X
Developmental delays		X				
No somites		X				
Non-detachment of the tail		X				
No pigmentation		X				
Heart rate			X			
Hatching success				X	X	X
Malformations						X
Oedema						X
Eye/brain defects						X
Deformation of the spine						X
Light pigmentation						X

### Statistics

The data were analysed with *JMP*^*®*^ *11.2.0* (*SAS Institute Inc. 2013*). Data on mortality, hatching rate, malformations (all after 96 hpf), and developmental delays (after 24 hpf) were tested with a Likelihood Ratio *χ*^2^. For post hoc analyses, Fisher’s exact test was conducted and to account for multiple testing the Sequential Bonferroni–Holm correction was used. For the analysis of heart rate, the data was averaged per Petri dish and checked for normal distribution and homogeneity of variances. If those criteria were met, an ANOVA with a Tukey HSD test was conducted. Otherwise, the alternative was a non-parametrical Kruskall–Wallis combined with a Steel–Dwass-test.

To avoid inconsistency in the data analysis, all runs were analysed individually as some controls within sampling event sets were statistically significantly different from one another, especially concerning the endpoints ‘heart rate’ and ‘hatching success’.

### Calculation of ecological assessment

The general ecological assessment of each sampling site is based on statistically significant results obtained for endpoints, compared to the control. Endpoints would be regarded as significantly different, if at least two out of the three parallel runs showed statistically significant results. Therefore, data was not averaged by trial. The different endpoints (mortality, hatching success, developmental delays, malformation, heart rate) were rated equally. Therefore, one sampling site could obtain a maximum of five significances per sampling event, adding up to 20 for four sampling events in total. The defined categories of the ecological evaluation (very good, good, moderate, poor, and very poor) are based on the calculated percentages of significant differences vs. the respective controls per sampling site throughout four sampling events (for criteria see Table 1 and Fig. 1).

Percentages for developmental delays and malformation were calculated by dividing the maximum number of delay/malformation characteristics (see Table 2) that can potentially occur per treatment by the number that actually occurred multiplied by one hundred. Each characteristic was counted maximally once per individual.

### Chemical analysis of sediments

For the PCB and PAH analyses, sediments were treated according to the slightly modified standard protocols prEN 16167:2010 and prEN 16181:2010, respectively. Briefly freeze-dried, ground and sieved (< 2000 µm) sediment samples of 5 g were extracted and purified by pressurised liquid extraction (PLE) combined with an in-cell cleanup using an ASE-350, (*Thermo Scientific*, Dreieich, Germany). The 34 mL extraction cell was filled to capacity according to the following setup: one cellulose filter was placed at the bottom of the extraction cell, followed by 1.5 g pre-washed copper powder, a cellulose filter, 3 g silica powder and another cellulose filter. 5 g of dried and sieved sample was mixed with a sufficient amount of sea sand and carefully poured into the extraction cell, followed by a cellulose filter. The extraction was done twice, each with 40 mL of a mixture of iso-hexane, acetone and n-heptane (62:33:5; v/v/v). Subsequently a mixture with surrogate standards was added containing 31 13C-labelled PCBs and 16 deuterated PAHs. Sample extracts were combined and concentrated to 0.5 mL using a Büchi Synchore evaporator (BÜCHI, Konstanz, Germany) at 40 °C. Afterwards a GPC-cleanup (*Shodex CLNpakPAE 800 AC* 8.0 × 300 mm) with acetone as solvent was accomplished and the final sample volume was reduced to 0.5 mL.

The separation, identification and quantification of PCBs were performed using gas chromatography (*Agilent 7890B*, Waldbronn, Germany) coupled to triple quadrupole mass spectrometry (Agilent 7010B, Waldbronn, Germany). For the GC–MS/MS, a SGE HT8 column (50 m, 0.22 mm ID, 0.25 μm) was used. 2 µL (splitless) was injected at 280 °C with a purge flow of 50 mL/min. The oven was set to 80 °C (hold 2 min) to 170 °C at 30 °C/min to 300 °C at 3 °C/min (hold 9 min) to 350 °C at 60 °C/min (hold 2 min). Helium in a constant flow (1.7 mL/min) was used as carrier gas (transfer line 290 °C). A MS/MS detector with MRM using electron ionisation (El at 70 eV) was applied (source temperature: 230 °C; quad: 150 °C).

The separation, identification and quantification of PAHs were performed using gas chromatography (*Agilent 6890N*, Waldbronn, Germany) coupled to triple quadrupole mass spectrometry [*Agilent 5975B*, Waldbronn, Germany, with *Evolution* (*Chromtech*)]. For the GC–MS/MS, a *Rxi*^*®*^-PAH column (40 m, 0.18 mm ID, 0.07 μm) was used. 2 μL (pulsed splitless) was injected at 100 °C with a pulse pressure of 4 bar, pulse time of 1.5 min and a purge flow of 40 mL/min. The oven was set to 70 °C (hold 1 min) to 190 °C at 15 °C/min to 300 °C at 2 °C/min (hold 3 min) to 340 °C at 20 °C/min (hold 5 min). Helium in a constant flow (1.0 mL/min) was used as carrier gas (transfer line 320 °C). A MS/MS detector with MRM using electron ionisation (El at 70 eV) was applied (source temperature: 230 °C; quad: 150 °C).

For quality assurance the certified reference sediment SRM 1941b [20] was extracted and analysed within each sample run. The marine sediment SRM 1941b is certified for 26 compounds (PCBs, organochlorine pesticides and PAHs). The recovery for all substances was always in an acceptable range of 80–110%.

For the (heavy) metal analysis (see also [21]), 1–10 g freeze-dried and ground sediment samples with residual pore water contributing to < 10% of the soil concentrations were used. After microwave digestion, the total metal concentrations were measured in nitric acid.

All analyses of PAHs, PCBs and (heavy) metals were conducted accordingly to European (EN) or German (DIN) standard test protocols without analytical replication.

For the TOC analysis, the freeze-dried sediment samples were analysed according to DIN EN 13137 [22] with an *ELTRA Helios C/S Analyser* (*ELTRA GmbH*, Haan, Germany) after dry combustion with subsequent infrared detection. The samples were acidified with hydrochloric acid to release inorganic carbon prior to the IR-detection. Particle sizes were determined using a cascade of sieves with mesh sizes between 2 000 µm and 20 µm. Dry-sieving applied to fractions of ≥ 630 µm, whereas for smaller fractions wet-sieving in an ultrasonic bath was used (procedure according to [23]).

Reported sediment concentrations and proportions are based on dry weight sediment (dws).

## Results

### Pretrials without sediment

No effect was detected in embryos from any sampling site regarding all evaluated endpoints (data not shown).

### Trials with sediment

In this case study, mortality was of no concern. Only twice across all sampling events, sites and runs mortality of the embryos was slightly but significantly elevated, compared to the control. Apart from the occasional observations, mortality was not statistically differing from the negative control and is, therefore, not considered to be of relevance in this case (data not shown).

The variation in heart rate and hatching success seemed mainly to be linked to the extent of developmental delays with low heart and hatching rates in developmentally retarded individuals. Consequently, those results are not discussed in further detail. Additionally, heart rates tend to vary and may be regarded as a rather sensitive endpoint towards external influences, e.g. temperature, and thus, should be interpreted with care at all times [24, 25]. As a consequence, the main focus is placed on the endpoints ‘developmental delay’ and ‘malformation rate’. Rates of developmental delays were exceeding effects regarding malformations, by far.

Sediments from the river Nidda were slightly organic, mainly consisting of fine and/or coarse gravel. The proportion of organic material soared at N3 and then slowly decreased in flow direction until site N6 (TOC is shown in Table 3 and for particle size distribution see also Table 4). The water appeared clear and odourless, and contained only a very small amount of suspended matter.

**Table 3:** Chemical analytics of sediments from the main sampling sites concerning PAH, PCB and (heavy) metal contents, as well as the proportion of total organic carbon (TOC) exemplarily shown for the sampling event in winter/spring 2016

	Main sampling sites													
	N1	N2	N3	N4	N5	N6	H1	H2	H3	H4	U1	U2	U3	U4
Benz[a]anthracen (mg/kg)	0.150	0.104	0.163	0.170	0.065	0.175	0.026	0.106	0.061	0.043	0.315	0.174	0.222	< LOQ
Benzo[a]pyrene (mg/kg)	0.088	0.086	0.166	0.155	0.074	0.186	0.034	0.117	0.079	0.055	n.a	0.208	0.219	0.008
Fluoranthene (mg/kg)	0.370	0.232	0.369	n.a	n.a	n.a	0.084	0.263	0.119	0.087	0.712	0.431	0.749	0.009
Fluorene (mg/kg)	0.011	0.016	0.024	n.a	0.013	0.024	0.005	0.013	0.007	0.006	0.014	0.009	0.023	0.002
Phenanthrene (mg/kg)	0.134	0.111	0.222	n.a	0.099	0.289	0.062	0.142	0.067	0.042	0.188	0.122	0.349	0.009
Pyrene (mg/kg)	0.245	0.152	0.252	0.212	0.090	n.a	0.059	0.200	0.091	0.063	0.453	0.281	0.462	0.005
PCB 28 (µg/kg)	0.014	0.110	0.266	0.420	0.193	0.410	0.020	0.030	0.125	0.137	0.072	0.076	0.052	0.103
PCB 52 (µg/kg)	0.029	0.464	0.486	1.219	0.359	0.586	0.028	0.286	1.390	0.194	0.099	0.272	0.280	0.169
PCB 101 (µg/kg)	0.262	3.932	1.594	2.824	0.946	2.835	0.081	2.043	3.486	0.703	0.436	1.008	1.223	1.006
PCB 138 (µg/kg)	0.498	9.366	5.504	5.646	2.785	6.763	0.169	3.618	4.430	2.479	1.093	3.116	1.966	1.869
PCB 153 (µg/kg)	0.645	11.98	6.248	6.365	3.435	8.561	0.268	4.321	4.142	2.894	1.469	3.437	2.175	2.714
PCB 180 (µg/kg)	0.456	10.34	5.425	4.089	2.583	5.611	< LOQ	2.597	2.252	2.009	0.860	1.920	1.317	1.632
Arsenic (mg/kg)	< LOQ	7.626	< LOQ	< LOQ	5.815	10.83	< LOQ	5.935	< LOQ	< LOQ	12.25	10.87	18.81	60.64
Cadmium (mg/kg)	0.315	0.527	0.252	0.296	0.388	0.572	0.220	0.344	0.320	0.425	0.385	0.263	0.719	1.205
Chromium (mg/kg)	173.8	199.4	113.9	103.6	123.9	101.7	136.9	113.9	114.5	128.1	62.42	66.18	84.61	86.88
Copper (mg/kg)	31.80	52.65	26.01	20.80	29.68	32.64	18.21	22.42	21.52	29.35	26.94	26.94	56.69	52.12
Lead (mg/kg)	27.96	45.52	30.83	24.29	29.50	44.02	20.15	21.29	20.38	25.13	38.39	62.23	121.3	137.0
Mercury (mg/kg)	0.083	0.096	0.180	0.107	0.152	0.156	0.052	0.043	0.067	0.140	0.065	0.083	0.115	0.248
Nickel (mg/kg)	112.9	171.8	84.53	72.73	87.15	71.51	116.9	110.3	86.95	102.8	52.81	52.42	82.89	71.47
Zinc (mg/kg)	142.7	224.9	155.9	132.0	219.5	529.5	103.6	120.1	151.7	202.0	133.9	222.8	426.3	1287
TOC (%)	0.540	0.552	4.409	3.113	2.136	3.281	2.191	1.515	3.907	3.922	0.816	5.253	0.454	0.663

*LOQ* limit of quantitation, *n.a* not analysed

**Table 4:** Particle size distribution from the main sampling sites exemplarily shown for the sampling event in winter/spring 2016 as measured by the particle size analyser Beckmann Coulter LS 200

	Particle size distribution (µm)				
	0–20	20–63	63–200	200–630	630–2000
N1	10.4	8.95	12.7	49.0	19.0
N2	19.8	3.9	4.4	32.8	39.1
N3	28.2	27.9	25.4	14.6	3.9
N4	24.7	25.2	25.3	20.5	4.2
N5	31.5	21.8	21.0	23.6	2.1
N6	21.3	17.0	20.7	22.5	18.5
H1	22.6	14.9	17.4	29.0	16.1
H2	13.9	13.9	14.7	22.2	35.4
H3	29.0	37.4	21.8	8.9	2.9
H4	36.6	32.5	18.9	10.2	2.1
U1	41.4	14.9	14.0	21.4	8.2
U2	29.2	23.7	21.4	18.7	7.1
U3	11.2	4.6	10.8	54.3	19.1
U4	25.3	7.7	9.3	23.4	34.3

**Fig. 2:**
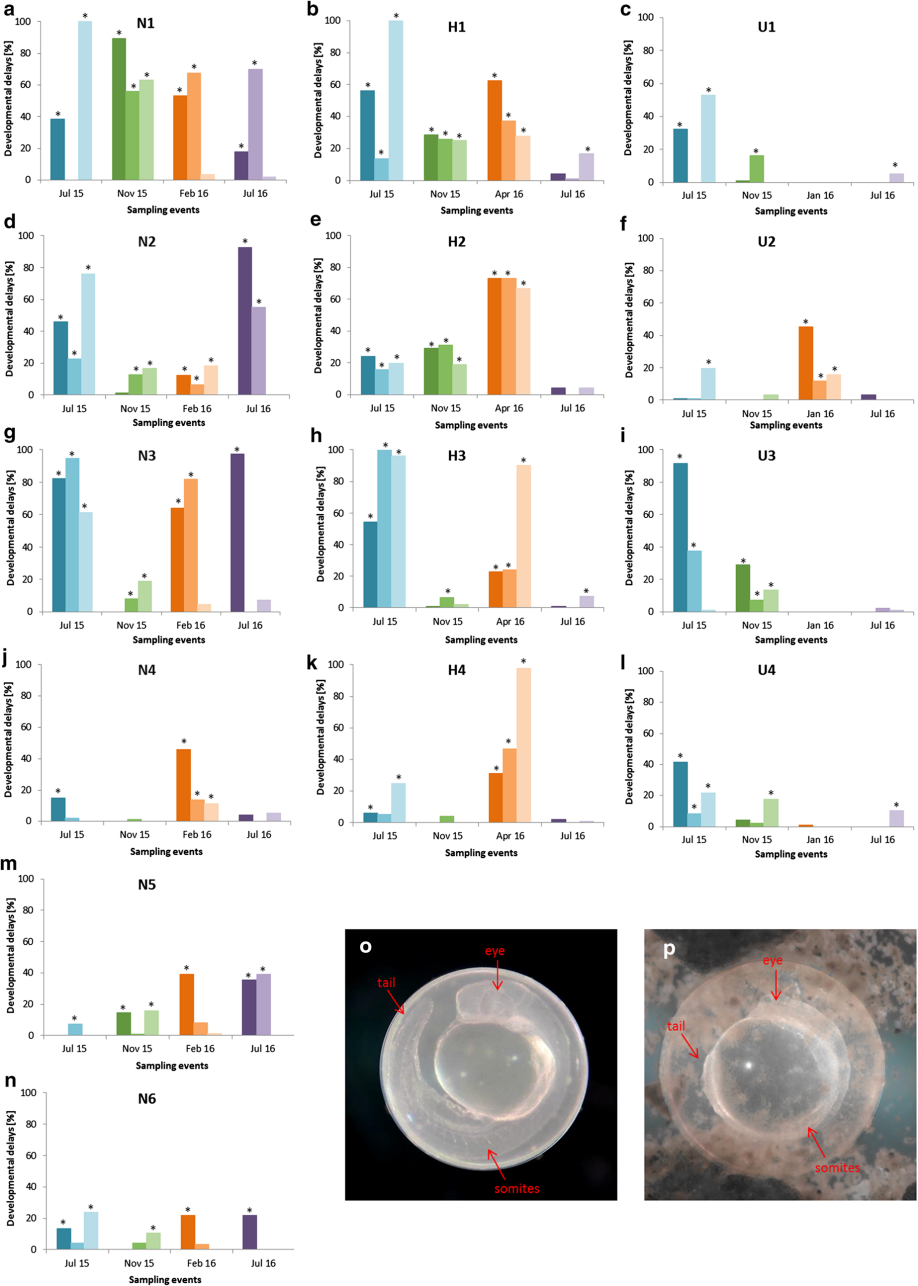
Developmental delays at 24 hpf (in %) for samples from the main sampling sites throughout all sampling events. **a**, **d**, **g**, **l**–**n** show results from the River Nidda, **b**, **e**, **h**, **k** from River Horloff and **c**, **f**, **i**, **l** from River Usa. Asterisks mark significant differences to control treatments (no developmental delays detected in controls; data not shown); Likelihood ratio *χ*^2^, Fisher’s exact test (*p* < 0.05), correction with sequential Bonferroni–Holm method *p* < *α*. Photograph o shows a control embryo at 24 hpf with detached tail, normally developed eyes and somites. Photograph p depicts a clearly retarded embryo lacking tail detachment and showing delay in eye and somite development

Results for developmental delays, malformation and hatching success in embryos developed in Nidda water and sediment showed a clear difference between the first three sampling sites (N1–N3) upstream and last three (N4–N6) downstream Nidda River. Embryos exposed to water and sediment from N1, N2 or N3, in particular, showed elevated rates of developmental delays, (Fig. 2a, d, g), as well as increased rates of malformations (Table 5), on a lower level, mainly ranging between 5 and 10%. By contrast, at downstream sites rates of developmental delays were considerably lower and malformations were hardly occurring. In this context, we only saw minor variations between years, since results from July 2015 and from July 2016 were largely consistent except for sampling site N5.

**Table 5:** Malformation rates at 96 hpf for main and additional sampling sites with three runs per time point

	Malformation rate (%)											
	July 15			November 15			January 16–April 16			July 16		
Control (N)	0	0	0	0	0	0	0	0	0	0	0	0
N1	2.42	0	7.14*	15.52*	12.10*	16.67*	2.0	12.10*	0.93	1.19	10.48*	0
N2	3.13	0	5.36*	3.57	7.26*	8.62*	1.85	0	0	15.74*	9.68*	1.56
N3	10.15	4.03	9.38*	6.25*	0.86	6.25*	9.00*	9.68*	1.67	11.11*	0	3.13
N4	0	2.34	0	3.91	2.68	0.83	9.52*	0	0.78	1.61	0.78	0
N5	0	0	3.23	7.03*	2.50	1.61	4.46	3.45	1.72	2.42	3.45	0
N6	3.23	0	7.81*	3.33	6.45*	3.13	3.85	0	0	2.34	0	0
Control (H)	0	0	0.78	0	0	0	1.56	0	0	0	0	0
H1	6.25	3.91	6.25	3.70	3.33	0	0.78	0	0	3.91	7.81*	3.13
H2	4.84	5.47	0	4.17	5.56	2.78	20.24*	0	8.33*	2.34	1.61	0.78
H3	2.42	13.79	21.0	0	0.83	0	0	0	0.81	2.50	3.45	9.17*
H4	0.81	0.78	0	0	0	0	0	2.34	10.94*	5.47	1.61	4.69
Control (U)	0	0	0	0	0	0	0	1.56	0	0	0	0
U1	3.70	1.61	3.13	0	0.93	0	0	0	0	0.78	4.69	0.81
U2	0	0	0.78	0	0	0.83	4.84	3.57	6.67	3.23	1.56	0.78
U3	15.74*	4.63	1.56	10.42*	1.61	4.31	2.50	0	0	3.91	6.25	3.13
U4	1.56	2.42	2.34	0.89	0	0.93	2.34	0	0	2.34	2.34	3.13

	March 17			June 17			September 17		
Control (N)	0	0	0	0	0	0	0	0	0
N1	2.08	0	0	2.17	0	2.08	0	0	0
N2.1	0	0	0	3.13	1.04	6.52	0	0	0
N2.2	0	0	0	1.04	3.13	0	0	0	1.14
N2.3	0	0	0	5.21	4.17	2.08	0	1.09	7.14*
N2.4	1.04	0	0	0	2.08	0	1.25	0	0
Control (H2)	0	0	0	0	0	0	0	0	0
H1	1.09	3.13	0	6.25	0	0	2.38	1.04	0
H2	0	1.04	1.04	0	0	0	0	0	3.13
H2-DE	2.34	0	1.04	0	0	0	1.04	3.13	2.08
H2-F	1.56	9.78*	1.04	13.54*	4.17	5.43	0	7.61	0
H2-B	21.09*	25.00*	20.83*	16.30*	14.58*	23.96*	4.17	3.26	1.04
H2.1	2.08	0	3.13	2.27	0	3.13	0	1.09	0
H2.2	5.21	1.09	7.29	2.08	0	0	0	0	1.0
H2.3	8.33*	4.17	1.04	0	0	2.08	0	1.04	0
Control (H2a)	0	0	0	0	0	0	1.47	0	1.32
H2a oh	3.13	0	0	3.57	3.13	5.43	2.63	0	0
H2a-DE	0.78	1.04	0	0	4.17	1.04	1.19	0	0
H2a uh1	3.13	0	0	4.17	5.21	3.13	0	25.00*	0
H2a uh2	11.96*	3.13	1.04	0	0	0	7.81	0	13.75*
H2a uh3	1.09	9.38*	0	4.17	0	0	0	21.25*	2.38
H2a uh4	0	1.04	0	0	0	0	0	18.75*	0

* Significant differences to control treatments; likelihood ratio *χ*^2^, Fisher’s exact test (*p* < 0.05), correction with sequential Bonferroni–Holm method *p* < α

The tests run with samples from sites downstream the industrial discharger resulted in higher rates of developmental delays and, concerning site N2.3, also of malformations, but with little variation between the sites for both developmental delays and malformations. No dilution effect in flow direction could be detected. At reference N1, effects were detected to a similar extent. Therefore, it was not possible pinpointing potential contributions of the industrial discharger to the unsatisfying to poor condition concerning the ecological assessment. The effects detected at N1 in 2017 were in line with findings from the previous years 2015/2016.

Horloff sediments were found to vary a lot with respect to particle size and organic content. The test included samples consisting of coarse sand lacking any organic matter (H2a-DE), samples with a high proportion of coarser and fine gravel (H1), almost sandy samples (H2), samples consisting solely out of organic matter (H2a oh–H2a uh 4, H3) and samples that were a moderate mixture of gravel and organic matter (H4). The organic matter itself was rather fine and muddied the water, especially in case of motion. Water from upstream sites appeared clear, whereas water from H2a oh downstream got increasingly murky and contained a high proportion of suspended matter. Water and sediment from those sampling sites, but from H3 in particular, were often characterized by a mouldy and putrid odour.

Concerning the results for the embryo toxicity, developmental delay rates showed a similar pattern by tendency compared to effects found in the Nidda River samples (Fig. 2). Embryos exposed to water and sediment from the two most upstream sampling sites H1 and H2 were significantly retarded in all three runs of the first three sampling events, whereas embryos exposed to samples from H3 and H4 showed such significance only for sampling events one and three. Particularly samples from H4 seemed to be the least conspicuous one. Results for the third sampling event in April 2016 were remarkable as the embryos were found to be significantly delayed in development in every run of each sampling site with rates between 23 and 98%.

In contradiction to results from the River Nidda, effects detected in July 2015 could not be seen in embryos exposed to samples collected in July 2016.

The malformation rates induced by River Horloff samples were rather low, mainly highest in samples from July 2015, but usually not exceeding 6% except for one aberration at H2 in April 2016 (Table 5).

**Fig. 3:**
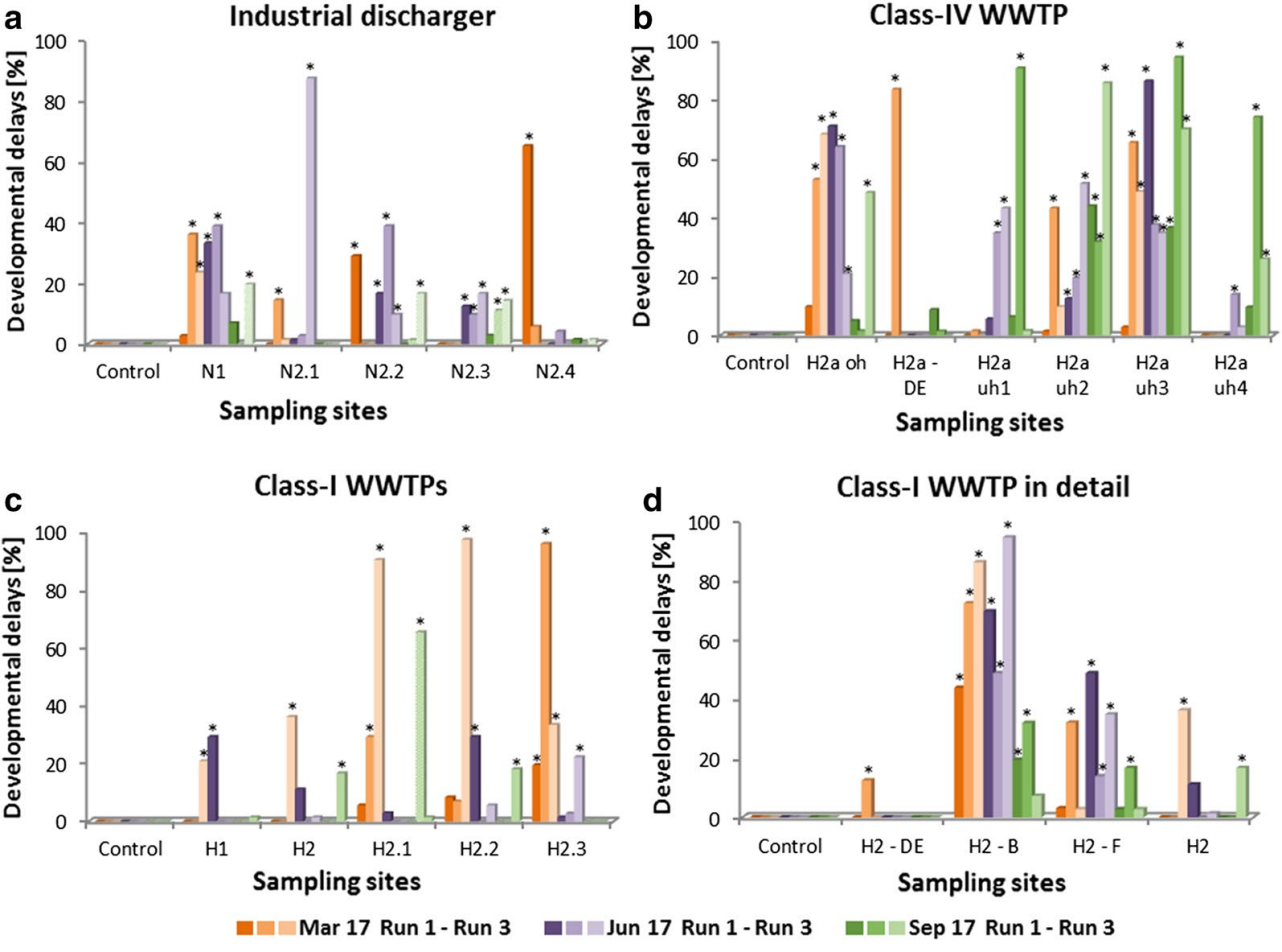
Developmental delays at 24 hpf for the additional sampling sites. Plot **a** shows results from upstream (N1) and downstream (N2.1–N2.4) of the industrial discharger at River Nidda. Plot **b** depicts results from upstream of (H2a oh), downstream of (H2a uh1–uh4) and directly from the efflux pipe (H2a-DE) of a class-IV WWTP at River Horloff. Plots c and d illustrate results from upstream (H1) and downstream (H2–H2.3) of the class-I WWTPs (**c**) and a more detailed resolution of the last class-I WWTP (**d**). Asterisks mark significant differences to control treatments; Likelihood ratio *χ*^2^, Fisher’s exact test (*p* < 0.05), correction with sequential Bonferroni–Holm method *p* < *α*

In 2017, less severe effects were detected in embryos exposed to samples from site H2 than in the years before. The subsequent downstream samples caused stronger effects than H2 itself. Although, there was a variation between runs within samples from the same site, variation between sites was rather low. Alike the sites downstream the industrial discharger at the Nidda, they could be considered to be in an unsatisfying to poor condition. More salient effects could be detected in the close-up sampling at site H2. Particularly samples collected from the basin area (H2-B) induced consistently high rates in both developmental delays (Fig. 3d) and malformation. Only samples from sampling event in September 2017 caused effects at a lower level, but still to larger extent than any other sample. In the samples from the area of limited mixture of river water and WWTP efflux (H2-F) similar effects could be detected, but already at a clearly reduced scale, whereas the efflux itself did not cause any effect, probably due to the lack of sediment.

The area around the class-IV WWTP was salient in the tests. Upstream and downstream samples induced high rates of developmental delays in the exposed embryos. Malformations occurred in considerably lower percentages, but more frequently than in the Nidda River and the regular class-I WTTP samples (H2). Particularly pronounced effects could be detected in embryos exposed to samples from H2a oh, H2a uh2, and H2a uh3, whereas samples collected directly from the efflux pipe (H2a-DE, just water, no sediment) hardly caused any effects (Fig. 3b).

Sediment and water appeared to be rather similar to that from upstream the Nidda, with fine and coarser gravel and clear, odourless water with a negligible portion of organic and suspended matter, except for U2 (Table 3).

Concerning the River Usa, developmental delays were especially induced by samples collected in July 2015, excluding sampling site U2 where water and sediment samples led to a higher delay rate for January 2016 but not July 2015. Elevated rates of developmental delay could be detected for samples from November 2015 at U3 and slightly at sites U1 and U4. Generally, sampling site U2 showed a diverging, almost opposite pattern compared to the other sites at River Usa (Fig. 2f). In the case of the Usa River, it might be linked to the proportion of organic material, as the amount of TOC was more than five times higher in comparison to the other Usa sites (Table 3). In relation to the rivers Nidda and Horloff developmental delays were induced at a considerably lower level.

Concerning malformations, only water and sediment from sampling site U3 induced slightly significant effects, whereas effects at the other sites were barely evident.

## Discussion

### General remarks

In general, our data has shown that water and sediment from sampling sites upstream the rivers induced more frequent effects at considerably higher levels in exposed embryos than downstream, in particular concerning developmental delays; an observation that was not expected in the first place. Compared to other studies carried out with samples from larger German rivers including Neckar [19, 26], Rhine [27] and Danube [28] that detected relatively high to very high embryotoxic potentials in sediments, our results reveal effects on sublethal levels, in particular. Regarding those sublethal effects, annual variations occurred. Comparing results from the first and the second sampling cycle for the sites N1, H1 and H2, a decrease in developmental delay and malformation rates were detected. Regarding seasonality, results from samples collected from the Nidda in February and Horloff in April 2016 show consistently high rates of developmental delays. The same applied to samples collected from rivers Horloff, Usa and the upstream Nidda sites for July 2015. Generally, in field studies, seasonal variation may be an influencing factor also reflected in matrix composition, but is expected to be less pronounced in sediments than in water, as sedimentation is a rather slow process. But seasonal variation is the reality organisms face in their environment. *Danio rerio* embryos themselves did not experience additional stress that may be attributed to seasonality, since they were exposed under controlled laboratory conditions.

The physicochemical parameters (see Table 6) revealed that all three rivers suffer from moderate nitrogen pollution, since they all run through intensely agriculturally used areas. However, nitrate concentrations were mostly below the environmental quality standard of 50 mg/L set by the European Union (Council Directive 91/676/EEC [29] ), except for the Usa (U2–U4) in November 2015, the Horloff in April 2016 (H2) and the Nidda (N2–N5) in July 2017, whereas concentrations for nitrite, ammonium and phosphate constantly exceeded limits for a good ecological status that is postulated as goal in the European Water Framework Directive (Directive 2000/60/EC) at all sampling sites. The high concentrations of nitrogen compounds in those surface waters reflect the intense agricultural use of land within their drainage and very likely originate from fertilisation on a regular basis. Studies on the effects of nitrate and nitrite on early developmental stages in fish have shown that nitrate is considerably less toxic than nitrite and even for nitrite high concentrations are needed to induce effects in embryos or larvae [30, 31]. Luo et al. [30] set the no observed effect concentration (NOEC) regarding larval growth in rare minnow (*Gobiocypris rarus*) at 19.95 and 13.33 mg/L for nitrate and nitrite nitrogen, respectively. The highest concentrations measured in this study were 23 mg/L for nitrate and 0.2 mg/L for nitrite nitrogen. Except for a single occasion when high nitrate nitrogen concentration (> 20 mg/L) coincided with pronounced effects in embryo development (H2 downstream the class-I WWTPs, April 2016), physicochemical parameters did not seem to be a driving factor for observed embryotoxicity—in particular concerning the fact that, generally, the physicochemical parameters tended to fall off in quality in downstream direction, whereas embryotoxicity had the tendency to decrease in parallel. This was in accordance with the pretrial findings, in which surface water alone did not induce any effect in zebrafish embryos. Thus, it is very likely that, in this case, hydrophilic substances that were primarily present in the water column were irrelevant for embryotoxicity. Instead, it must be assumed that factors related to the sediment were responsible for the effects observed. On the one hand, lipophilic substances that tend to bind to particles and accumulate in the sediment and, on the other hand, substances that may be solved in pore water might be of importance. Hollert et al. [19] compared the effects of pore water and sediment on zebrafish embryos and detected differences in severity of effects. Sediments induced higher embryotoxicity than corresponding pore waters concluding that the bioavailability of particle-bound lipophilic substances was considerably higher than previously assumed.

**Table 6:** Physicochemical parameters measured at the main sampling sites at the time of sampling

	Main sampling sites													
	N1	N2	N3	N4	N5	N6	H1	H2	H3	H4	U1	U2	U3	U4
pH														
July 15	7.46	7.86	7.37	7.43	7.62	7.81	7.79	7.91	7.77	7.84	7.09	7.92	8.20	7.75
November 15	7.56	7.99	7.50	7.74	7.77	7.77	7.43	7.83	7.95	7.84	7.10	7.50	7.40	7.66
January–April 16	6.98	6.98	6.73	7.27	7.11	7.37	7.19	7.21	7.69	7.98	7.00	7.31	7.10	7.35
July 16	7.85	7.73	7.36	7.36	7.58	7.62	7.76	7.30	7.54	7.36	7.64	7.85	7.96	7.73
Nitrate [NO_3_–N] (mg/L)														
July 15	1.20	1.40	1.70	2.70	2.60	2.70	0.50	1.70	5.00	2.80	2.50	5.60	3.60	3.70
November 15	2.00	4.00	8.00	11.0	9.00	12.0	2.00	5.00	7.00	5.00	4.00	23.0	14.0	21.0
January–April 16	7.00	13.0	8.00	2.71	2.26	15.0	7.40	> 20.0	0.70	0.90	3.40	4.20	3.90	4.40
July 16	8.10	10.6	12.2	12.0	10.9	8.10	4.30	4.40	7.03	3.80	3.90	5.50	4.60	3.90
Nitrite [NO_2_–N] (mg/L)														
July 15	0.01	0.01	0.10	0.08	0.09	0.04	0.01	0.02	0.05	0.06	0.05	0.02	0.04	0.13
November 15	0.01	0.01	0.07	0.09	0.07	0.05	< 0.003	0.02	0.04	0.03	0.01	0.02	0.03	0.13
January–April 16	0.02	0.02	0.02	0.004	0.01	0.05	0.03	0.03	0.04	0.03	0.04	0.06	0.05	0.11
July 16	0.01	0.02	0.20	0.04	0.04	0.04	0.02	0.03	0.05	0.06	0.04	0.01	0.06	0.13
Ammonium [NH_4_–N] (mg/L)														
July 15	0.06	0.04	0.26	0.08	0.14	0.07	0.05	0.05	0.22	0.16	0.09	0.04	0.17	0.18
November 15	< 0.04	0.04	0.67	0.34	0.23	0.21	< 0.04	0.10	0.16	0.10	< 0.04	< 0.04	0.07	0.27
January–April 16	< 0.04	< 0.04	0.12	0.08	0.10	0.13	0.05	0.05	0.06	0.07	< 0.04	0.09	0.12	0.20
July 16	< 0.04	0.06	0.68	0.04	0.05	0.07	< 0.04	0.15	0.11	0.10	0.07	0.05	0.16	0.09
Phosphate [PO_4_–P] (mg/L)														
July 15	< 0.05	0.05	0.17	0.26	0.30	0.32	0.05	0.12	0.16	0.22	0.06	0.27	0.13	0.28
November 15	0.05	0.05	0.13	0.28	0.37	0.26	< 0.05	0.20	0.16	0.17	0.10	0.22	0.17	0.28
January–April 16	< 0.05	< 0.05	0.10	0.02	0.05	0.16	0.07	0.12	0.19	0.19	0.20	0.25	0.27	0.22
July 16	0.07	0.08	0.12	0.24	0.22	0.27	0.08	0.16	0.17	0.25	0.10	0.32	0.20	0.19
Chloride [Cl^−^] (mg/L)														
July 15	8.0	10.0	28.0	73.0	86.0	> 200	5.0	18.0	108	106	29.0	91.0	120	> 200
November 15	9.0	17.0	61.0	85.0	83.0	256	8.0	31.0	86.0	85.0	28.0	71.0	96.0	824
January–April 16	11.0	25.0	22.0	35.0	29.0	83.0	< 2.5	< 2.5	20.0	25.0	22.0	34.0	34.0	270
July 16	11.0	14.0	40.0	39.0	46.0	194	7.0	23.0	80.0	96.0	47.0	67.0	69.0	> 200

The third sampling campaign was conducted in January (Usa), February (Nidda) and April (Horloff) 2016

Polycyclic aromatic hydrocarbons (PAHs), polychlorinated biphenyls (PCBs) and (heavy) metals are substances that accumulate in sediments and are all well-known to impact embryonic development and causing malformations, partially in even environmentally relevant conditions (e.g. [32–35]). To unfold their toxic potential, those substances must be bioavailable. Bioavailability is often achieved by binding to organic particles [36]. In that context, a modelling approach correlated zebrafish egg survival with the amount of i.a. organic matter in the sediment. With increasing proportion of organic matter, survival of the embryos decreased. However, particle size distribution was of no concern [7]. On the other hand, Perrichon et al. [34] revealed an opposing relationship: with increasing amounts of organic matter in sediments hydrophobic substances, particularly PAHs, have been reported to be less bioavailable and, therefore, embryotoxicity decreased. A third study [37] found high amounts of organic matter in sediment to cause developmental delays in the first 24 hpf that could not be detected afterwards. In our case, mortality was rarely present and was definitely not influenced by the proportion of organic matter. We observed embryos that were considerably developmentally retarded at 24 hpf but caught up rapidly, in a way that they could not be morphologically distinguished from ‘normally’ developing embryos at 72 hpf, at the latest. However, those findings occurred independently from the sediment’s characteristics, including TOC, and could not be directly linked to the amount and distribution pattern of PAHs, PCBs and (heavy) metals in the sampled sediments.

### Nidda

Unexpectedly, there was an obvious separation of an upstream (N1–N3) from a downstream (N4–N6) Nidda sampling set, with the downstream samples causing fewer effects than upstream ones. The improvement of embryonic health downstream may simply be due to dilution of pollutants, although three class-III and IV WTTPs discharge along the way, whereas upstream, only a single class-IV WWTP impacts the river. The results for N2 and N3 were nearly on the same level. The ecotoxicological quality of sediment and water at site N1 seemed to be a little ‘better’, in total, but the induction of developmental delays was more consistent throughout the seasons (Fig. 2).

Such results obtained for the upper regions of a small river demand an explanation. Possible reasons for the observed effects could be two storm water overflow basins (abbreviated SOBs in the following) located about 1 and 2.5 km upstream of N1 which collect road run-off during heavy rainfall events and discharge into the Nidda River. How discharges of SOBs negatively influence development in zebrafish embryos was shown by a field study [38] conducted at the Argen River, Southern Germany. The authors observed elevated mortality, malformation and developmental delay rates, as well as a decreased hatching success. Substances like PAHs, that i.a. stem from fossil fuels or originate from processes of incomplete combustion and thus are present in road run-off are organic, hydrophobic, environmentally persistent substances that tend to bind to sediments and therefore accumulate over time [39]. Considering our pretrial data which showed no effects in zebrafish embryos in the absence of sediments, the conclusion that sediment-bound substances are a likely reason for the results of our study is justified. PAHs, for example, are known to be able to pass through the chorion [40] causing adverse effects in fish embryos at higher concentrations, like oedema, reduction in cardiac function, reduced body length and eye defects [34, 41–45]. The same applies to polychlorinated biphenyls (PCBs) depending on their structure and mode of action. Whereas the LOEC for the coplanar PCB 126 is 37 µg/kg dws (dry weight sediment) for mortality and growth, and 176 µg/kg dws for larval abnormalities like oedema and skeletal deformities, the non-coplanar PCB 153 did not induce any effects in concentrations as high as 1350 µg/kg dws [46], indicating that CyP450-induced bioactivation may be an issue here. In a study from Hollert et al. [19] reduced and delayed hatching was reported for embryos exposed to sediments of a Neckar tributary (Germany) which was contaminated with PCB 138 (55 µg/kg) and PCB 153 (68 µg/kg). Although PAHs and PCBs were present in sediments from all three rivers examined, concentrations were considerably lower than in the studies mentioned above and usually considerably below the annual average EQS of 20 µg/kg [PAHs] set by the European Union [47, 48] (see Table 3). Nonetheless, they might be a factor that contribute to our findings and therefore should be taken into account. Furthermore, the dammed water reservoir located 5 km upstream N1 in the uppermost stretch of the Nidda River could have an influence on the N1 site. It can be presumed that water is occasionally drained from the reservoir and that the drainage carries all the substances with it that accumulated in the bottom water layer over time [49]. Even though considerable embryotoxicity was observed already at N1, the situation downstream towards site N2 became more critical. Although results from sites downstream the industrial discharger located in this area showed some variation (N2.1, N2.4), the embryotoxicity observed here has to be regarded amplified. In contrast, the WWTP downstream of N2 did not seem to have an additional adverse effect. In general, the four WWTPs along the studied course of the Nidda River did not seem to influence results in a prominent way.

### Horloff

In comparison to the Nidda River, the Horloff River even seems to be in a worse condition regarding developmental toxicity in fish embryos. The lack (H2-DE) or decrease (H2a uh1-DE) of effects in samples taken directly from the efflux pipe may be ascribed to absence (H2-DE) or characteristics (H2a uh1-DE) of the sediment: sediment collected from the efflux pipe at H2a uh1 consisted only of coarse sand without any organic components. It is very likely, that this type of sediment is not capable to adsorb organics [50] that may cause embryotoxicity. Although, in our study, the amount of organic matter did not correlate with the intensity of the observed effects; its presence as a particle sink seem conducive.

The results encompassing the several class-I WWTPs, on the one hand, and the class-IV WWTP, on the other hand, had similar implications. Samples inducing the severest effects were those not directly downstream the effluent, but those a little further along the river course (H2.1, H2a uh2, H2a uh3). Substances might need some time to sediment and do so increasingly in zones of lower flow velocity [49]. Another clear hint pointing in this direction is the results obtained from H2-B samples collected in the low velocity basin area close to the effluent. H2-B sediment induced consistently effects on a high level and more pronounced than in any other sample. Those effects seem comparable to findings in the lower Neckar region (Germany). Sediment samples collected from a less drained conservation area induced a higher embryo toxicity than samples directly from the Neckar, where sediments get permanently shifted [26]. Although, there is clear indication that discharges of the WWTPs influence embryotoxicity at the Horloff River, the results have to be regarded with suspicion as even samples from the uppermost site caused effects. Further to the mouth of the Horloff River entering the Nidda, the tendency of improved conditions downstream is reflected in the results obtained for site H4. Similar to the situation at the downstream sites of the Nidda River, there might be a slight dilution effect, as three ditches enter the Horloff between H3 and H4. Furthermore H4 is located between two renaturation areas that might have positive influence on self-purification processes and therefore, support mitigating effects.

### Usa

Regarding embryotoxicity, a similar pattern of results (lower embryotoxicity downstream than upstream) occurred in the Usa river, showing that there was already a basal contamination existing upstream the discharge of the most upstream WTTP. However, the recorded effects were less severe than in Nidda and Horloff. The WWTPs along the Usa all include the same purification steps and are almost of similar size (44,000–50,000 pe). Analogously, they could not be linked directly to the effects observed: results for U1, U2 and U3 were on the same level and, downstream the third WWTP, even a decrement in effects was found. Discharges of mineral spas, however, enter the Usa further downstream, which was reflected in high values for chloride and conductivity in the U4 water samples. Although zebrafish in the wild are well known for their tolerance towards a broad spectrum of environmental conditions [51–55], measures for conductivity beyond 3000 µS/cm at three out of four sampling events exceeded the usual values of up to 271 µS/cm in natural habitats [51] and our own housing conditions (260–350 µS/cm), by far. Interestingly, that did not affect the development of zebrafish embryos adversely, at all. The mineral spa discharges also make for the elevated levels of (heavy) metals measured in the sediment. Arsenic and zinc, in particular, exceeded EQS, considerably [47]. Arsenic is a metalloid that is known to be toxic to a wide range of organisms. In *Danio rerio* embryos, arsenic can cause i.a. spinal deformations, cardiac dysfunctions, altered cell proliferation, reduced growth, delayed hatching and increased mortality [56]. Similar effects can be induced by zinc in embryos of different fish species, including vertebral deformations, malformations of the eyes, oedema, reduction in size, fragile egg shells and irregular hatching [57–59]. Despite the increased contamination of (heavy) metals downstream the mineral spa discharges, U4 samples induced effects on considerably lower level compared to U1–U3 and was classified as ‘good’ in the ecological assessment. Low TOC might have contributed to that surprisingly good result, due to the lack of organic particles binding metals enhancing their bioavailability.

## Conclusions

Basically, in field studies like ours, it is often a difficult task to identify unambiguous effect patterns. Even more, it is rather impossible to pin definite cause–effect relationships, as a natural system is subject to various influences, biological and chemical processes. Abundant substances, from natural or anthropogenic sources, do not occur isolated from each other but in mixtures leading to a simple concentration addition or even feature more complex synergistic or antagonistic potentials. And although the circumstances of this case study applied specifically to the Nidda catchment system, four aspects may be considered to be able to be generally extrapolated to other river systems in regard to potential effects in biota:

1. Despite the fact that dischargers increase along the course of a river, it does not necessarily mean detectable biological responses to increase in the same way. On the one hand, there might be other sources beside specific point sources, e.g. diffuse contamination from agriculture, etc. that account for a basal pollution, influencing upstream zones adversely. On the other hand, dilution effects increase with increasing water quantities downstream and may mitigate negative impacts. Both factors might contribute to an outcome contrary to potential previous expectations.
2. Furthermore, our results show that chemical analyses of lead substances might not be sufficient to explain the effects induced in biota. Biomarker and bioassays, like the FET, help to bridge the gap between chemical measurements and biomonitoring in the field. They contribute to a better understanding of ecotoxicological potentials and shine a light on issues caused by mixture toxicity.
3. However, mortality was not an issue in this study, embryotoxic potentials based on sublethal effects clearly were. Thus, our results emphasise the importance of taking into account effects on subtle levels to obtain a realistic picture of pollution consequences.
4. This study should be taken as another reminder that sediments have to be taken into account for the biological assessment of ecosystem quality. Otherwise, it will not be manageable to reliably check for the ‘good ecological status’ of streams demanded by the European Water Framework Directive.

## Abbreviations

FET: fish embryo test
dws: dry weight sediment
EQS: environmental quality standards
hpf: hours post fertilisation
NOEC: no observed effect concentration
PAHs: polycyclic aromatic hydrocarbons
PCBs: polychlorinated biphenyls
REACH: Regulation (EC) No. 1907/2006 concerning the Registration, Evaluation, Authorisation and Restriction of Chemicals
SOB: storm water overflow basin
TOC: total organic carbon
WFD: Water Framework Directive (Directive 2000/60/EC)
WWTP: wastewater treatment plant
